# Reply to “Understanding the Role of Fungi in Chronic Wounds”

**DOI:** 10.1128/mBio.02033-16

**Published:** 2016-12-06

**Authors:** Lindsay Kalan, Sue E. Gardner, Elizabeth A. Grice

**Affiliations:** a Department of Dermatology, University of Pennsylvania, Perelman School of Medicine, Philadelphia, Pennsylvania, USA; b University of Iowa, College of Nursing, Iowa City, Iowa, USA; c Department of Microbiology, University of Pennsylvania, Perelman School of Medicine, Philadelphia, Pennsylvania, USA

## REPLY

We recently reported the fungal “mycobiome” and its association with outcomes in a longitudinal prospective cohort study of 100 patients with diabetic foot ulcers (1). We appreciate the letter to the editor by Malone and Dickson (2) and are pleased that our findings generated interest and discussion among basic scientists and clinicians.

The technique used to collect a sample for microbiome analysis is of great importance. All downstream processing and analysis relies on an accurate and reliable sample. Malone and Dickson are correct in that other groups advocate using more-invasive methods, though rigorous, peer-reviewed evidence to suggest the superiority of those invasive techniques is lacking in the literature. Interestingly, we noted that Malone authored a paper, “Deep Wound Cultures Correlate Well with Bone Biopsy Culture in Diabetic Foot Osteomyelitis” (3), in which the results seemingly contradict the argument presented in the letter. Nonetheless, we are most appreciative for the opportunity to further expand on our reasons for employing the Levine technique for collecting swab specimens.

Levine’s technique is different than other swab techniques in that it samples fluid from deep tissue layers, similarly to aspiration of wound fluid. In this method, the wound is cleansed with nonbacteriostatic saline and a swab is rotated over a 1-cm^2^ area of viable, nonnecrotic wound tissue for 5 s with sufficient pressure to mechanically disrupt the polysaccharide matrix associated with biofilms and sample organisms. Our reasons for employing Levine’s technique, as opposed to obtaining tissue, for specimen acquisition, are as follows.

(i) Tissue specimens contain large amounts of “contaminating” human DNA that interferes with bacterial DNA isolation, 16S rRNA gene amplification, and metagenomic sequencing. Although previous studies of human wound microbiota have employed curettage as the method of specimen acquisition, Han et al. (4) reported that human DNA or blood in the curettage specimen interfered with amplification of 16S rRNA genes, resulting in loss of 25% of the samples. We have experienced similar failures when working with punch biopsy specimens.

(ii) Culture results based on Levine’s technique are accurate (5). Malone and Dickson are correct in that previous work by our team showed that Levine’s swab technique is accurate with respect to measuring microbial load and diversity. While other studies comparing swabs to tissue cultures (6–9) report inconsistent results with respect to the accuracy of swabs, those studies suffered from numerous methodological problems. Perhaps the most serious methodological problem is that the studies did not describe specific techniques used to collect swab specimens (7, 9, 10). The levels of accuracy of swabbing techniques vary according to wound bed preparation, area of the wound sampled, and duration of sampling. These variables may have been key confounding factors in studies comparing swab specimens to tissue specimens from “discrete” wound locations. For example, Frank et al. (11) found poor concordance between swabs and tissue specimens, both of which were analyzed with 16S rRNA gene sequencing, but the swabs were collected using a “global” surface technique (i.e., the entire wound surface was swabbed), while tissue biopsy specimens were collected from a small single, discrete wound location. Moreover, their results were confounded by the fact that the DNA extraction techniques used for swab specimens were different than those used for tissue specimens. A critical aspect of any well-designed microbiome study is to keep methods (sampling, DNA extraction, PCR, sequencing) consistent across the study, as each method for these procedures is associated with its own inherent biases (12).

(iii) In the interest of the patients participating in the study, longitudinal sampling of chronic wounds must employ the least invasive specimen technique possible. Taking wound tissue every 2 weeks from a chronic wound poses a substantial risk to subjects who already have nonhealing wounds. We would never advocate for this type of sampling, nor would any Institutional Review Board view this favorably. Moreover, in our experience, study protocols requiring the acquisition of wound tissue resulted in the loss of 28% of potential subjects because they understandably object to having tissue removed from their wound (13).

To the second point of Malone and Dickson that we reported only on fungal communities, in fact, we reported on bacterial communities as measured using 16S rRNA gene sequencing, which was referenced and discussed several times in the paper (14). In addition to discussing this separately published study, we reported in the text an analysis of the two data sets together. We evaluated the community stability and dynamics between fungal and bacterial communities, which is detailed in Fig. S3. We also evaluated associations between fungal and bacterial phylotypes, which is illustrated in Fig. S6.

Because Malone and Dickson suggest that by sequencing the ITS1 and 16S rRNA genes together, one might obtain microbial load data, we want to emphasize that these approaches produce compositional data and are not appropriate for determining absolute abundances. For evaluating relative abundances of bacteria, fungi, archaea, and viruses within a sample, the more appropriate technique is metagenomic shotgun sequencing. Even with metagenomic sequencing, relative abundances within a sample are not synonymous with the total bioburden within the tissue. However, to categorize taxa as “major or minor players” based on relative abundance determined by amplicon-based sequencing would be shortsighted in our opinion, as relative abundance does not necessarily equate to pathogenesis or virulence.

Finally, based on the rationale of Malone and Dickson, one could argue that the clinical relevance of studies considering only bacterial communities is lost because fungal communities are omitted. We of course do not believe this but hope that our work has illuminated the complexity of the microbial communities inhabiting chronic wounds and look forward to additional studies that improve our understanding of polymicrobial contributions to impaired wound healing. Unfortunately, basic research is not always immediately translatable and/or interpretable in clinics. However, it is basic research that will provide deeper understanding of the microbial mechanisms governing wound healing, which we are optimistic one day will inform better management and treatment strategies for the millions of patients suffering from chronic wounds.
